# Thresholds for minimum clinically important difference, minimal important change and patient acceptable symptom state for the ACL‐RSI and the K‐SES in patients after anterior cruciate ligament reconstruction

**DOI:** 10.1002/ksa.70044

**Published:** 2025-09-04

**Authors:** Ramana Piussi, Jakob Lindskog, Rebecca Hamrin Senorski, Roland Thomeé, Kristian Samuelsson, Eric Hamrin Senorski

**Affiliations:** ^1^ Department of Health and Rehabilitation Unit of Physiotherapy, Institute of Neuroscience and Physiology, Sahlgrenska Academy University of Gothenburg Gothenburg Sweden; ^2^ Sahlgrenska Sports Medicine Center, Sahlgrenska Academy Gothenburg Sweden; ^3^ Sportrehab Sports Medicine Clinic Gothenburg Sweden; ^4^ Department of Orthopaedics Institute of Clinical Sciences, Sahlgrenska Academy University of Gothenburg Gothenburg Sweden

**Keywords:** evaluation, knee, psychometric, questionnaires

## Abstract

**Purpose:**

The aim of this study was to calculate and provide Patient Acceptable Symptom State (PASS) thresholds, Minimum Clinically Important Difference (MCID), and Minimal Important Change (MIC) values for the ACL‐Return to Sport after Injury (ACL‐RSI) scale and the Knee Self‐Efficacy Scale (K‐SES) in patients treated with ACL reconstruction.

**Method:**

Data were extracted from a rehabilitation specific registry, Project ACL. The registry prospectively collects patient‐reported outcomes (PROs). PASS, MCID and MIC were calculated using receiver operating characteristic (ROC) curve analysis and anchor‐based methods. Calculations were made for patients who completed the ACL‐RSI and K‐SES_18_ at follow‐up points 4 (K‐SES_18_ only), 8, 12 and 18 months post‐surgery.

**Results:**

A total of 704 patients aged 15–50 year, who underwent ACL reconstruction were included. The PASS thresholds increased over time, with AUC values indicating acceptable discrimination at all follow‐ups, especially at 18 months. The MCID values ranged from 1.6 to 4.1 for the K‐SES_18_ and 13.6–30.4 for the ACL‐RSI. The MIC values varied between follow‐ups, with negative or near‐zero values observed at certain intervals, particularly for K‐SES_18_ future.

**Conclusion:**

The PASS, MCID and MIC values for the K‐SES_18_ and the ACL‐RSI vary over time. Practical thresholds are provided for clinicians to better interpret scores and to determine if meaningful improvements have been achieved.

**Level of Evidence:**

Level III.

**Abbreviations**
ACL: anterior cruciate ligament
ACL‐RSI: anterior cruciate ligament return to sport after injury scale
AUC: area under the curve
CD: change difference
IKCD: International Knee Documentation Committee
IMP: improved
KOOS: Knee Injury and Osteoarthritis Outcome Score
K‐SES: Knee Self Efficacy Scale
MCID: minimum clinically important difference
MIC: minimal important change
NIMP: non‐improved
PASS: patient acceptable symptom state
PROs: patient reported outcome measures
ROC: receiver operation characteristic
SAS: statistical analysis system
SD: standard deviation
STARD: standards for reporting diagnostic accuracy

## INTRODUCTION

After a patient has sustained an injury and received appropriate treatment, the subsequent evaluation of the treatment results is an important step in evidence based medicine [22]. In the field of anterior cruciate ligament (ACL) injury, the recommended evaluation of treatment results includes clinical tests of muscle function, and patient reported outcomes (PROs) [25].

Different PROs are commonly used to evaluate treatment results. The responses are converted into total scores, which can then be analysed to determine whether there are statistically significant differences between groups [11]. However, statistical significance alone does not indicate whether the difference is meaningful to patients, nor does it reflect the size or clinical relevance of the difference [20]. In fact, with increasing sample sizes in orthopaedic research, even small and clinically irrelevant changes in PROs can become statistically significant [17]. To ensure that outcome interpretation reflects both statistical and clinical relevance, it is essential to report thresholds such as the Patient Acceptable Symptom State (PASS), Minimal Clinically Important Difference (MCID), and Minimal Important Change (MIC) [17]. A detailed description of PASS, MCID and MIC, and their interpretation, is presented in the Methods section (Table 1). These concepts help bridge the gap between statistical analysis and what actually matters to patients, supporting more meaningful interpretation of PRO scores in both clinical trials and routine care.

**Table 1:** Overview of concepts used for interpretation of PROs.

Concept	Definition	Clinical application
PASS	A threshold score on a PRO above which patients perceive their current knee function as acceptable [16].	If a patient's PRO score is above the PASS (e.g., >8.2 on the K‐SES_18_ present at 18 months), the patient's self‐efficacy is likely acceptable. If below, further follow‐up or tailored intervention may be needed. Can be used to screen individuals or evaluate recovery rates in a group.
MCID	The smallest difference in score between two groups of patients (e.g., improved vs not improved) that is considered clinically meaningful from the patient's perspective [4].	If a patient's K‐SES_18_ present score improves by less than the MCID (e.g., <2.3 points at 4 months), the change in self‐efficacy may not be perceived as meaningful by the patient, and tailored interventions might be needed. Can also be used in research to compare mean group differences between treatment approaches, e.g. standard rehabilitation vs rehabilitation with psychological support
MIC	The smallest within‐patient change in score over time that a patient perceives as important [27].	If a patient's K‐SES_18_ present score increases by more than the MIC (e.g., >2.1 points from 4 to 12 months), the patient likely perceives this as a meaningful improvement. Can be used during follow‐up to evaluate whether treatment has made a noticeable difference for the individual patient

Abbreviations: K‐SES_18_, knee self efficacy scale 18 item version; MCID, minimum clinically important difference; MIC, minimal important change; PASS, patient acceptable symptom state; PROs, patient reported outcomes.

Despite that numerous studies have reported PROs for patients after ACL injury [5, 6, 21] there are still questions with regard to interpretation of the actual scores. For patients who sustain an ACL injury, the two most widely used PROs [6] are the International Knee Documentation Committee Subjective Knee Form (IKDC‐SKF) and the Knee injury and Osteoarthritis Outcome Score (KOOS), for which the PASS, MCID and MIC thresholds have been calculated [16]. However, PASS, MCID and MIC have yet not been calculated for two other PROs commonly used to evaluate psychological outcomes in patients after ACL injury or reconstruction, that is, the ACL‐Return to Sport after Injury scale (ACL‐RSI) and the Knee Self‐Efficacy Scale (K‐SES).

Therefore, the aim of this study was to calculate and provide PASS thresholds, MCID and MIC for the ACL‐RSI and the K‐SES in patients treated with an ACL reconstruction.

## METHODS

Reporting followed the STARD checklist [3]. Data for the present study were prospectively collected from a rehabilitation outcome registry, Project ACL. The registry was established in 2014 and aims to improve the care of patients who suffer an ACL injury regardless of treatment. Data in Project ACL consists of results from muscle function tests and PROs. Data are collected prospectively with ACL injury or reconstruction as baseline, at pre‐surgery (in case of ACL reconstruction), 10 weeks, 4, 8, 12, 18 and 24 months, and every five years. At the time of follow‐up, patients are invited to perform muscle function test. Within 1 week from the test of muscle function patients are asked to respond to PROs on an online platform which automatically records patients' answers. Prior to participation in Project ACL, informed consent is collected. Ethical approval for the project was obtained from the Swedish Ethical Review Authority (registration number: 2020‐02501) and the Regional Ethical Review Board in Gothenburg, Sweden (registration numbers: 265‐13, T023‐17). Data from the present study was extracted from Project ACL in April 2024.

### Instruments

The PROs used in this study were the ACL‐RSI and the 18‐item version of the K‐SES (K‐SES_18_) including both present and future subscales, as well as two specific anchor questions.

The anchor questions, that patients are asked to respond to at each follow‐up in Project ACL, are formulated as follows:

(a) Do you perceive your current physical status as satisfactory? (yes/no).
(b) Do you think the treatment you received has markedly improved your condition? (strongly disagree/disagree/neither agree or disagree/agree/strongly agree).

The ACL‐RSI aims to measure patients' emotions, confidence, and risk appraisal of return to sport (RTS) after an ACL injury. In this study, the 12‐item version was used [28]. Each item on the ACL‐RSI was graded from 1 to 10, where 10 is the highest response, that represents the best possible psychological response towards RTS, that is, highest confidence and emotion, and lowest risk appraisal. The final score is calculated by summarising the total score of all items, highest score 120, and then normalising the score to a 10–100 scale [28]. The ACL‐RSI has demonstrated high internal consistency (*α* = 0.96), good construct and divergent validity and has been validated in a Swedish context [12, 29], although showed signs of multidimensionality and local dependence when assessed with a Rasch measurement theory [18].

The K‐SES_18_ aims to evaluate knee‐related self‐efficacy, that is, the belief in one's ability to successfully perform a physical task, such as to run or to jump. The K‐SES_18_ comprises 18 items divided into two subscales: present (14 items) and future (4 items) knee self‐efficacy. Each item is graded from 0 to 10, with 10 being the most positive response, which represent the greatest belief in successfully carrying out a given physical task. The results from each item are added and divided by the number of items to generate a mean value for each subscale. The K‐SES_18_ has been reported to have acceptable test‐retest reliability (ICC = 0.92 for both subscales), and acceptable construct validity to assess knee self‐efficacy in patients who suffer an ACL injury [1].

### Participants

Patients aged 15–50 years at ACL reconstruction, registered in Project ACL with one ACL injury, treated with reconstruction, who responded to both the K‐SES_18_ and the ACL‐RSI at any of Project ACL follow‐ups, and answered to both of the anchor questions at the follow‐up in which they respond to the K‐SES_18_ or the ACL‐RSI were eligible for inclusion.

For this study, demographic data and patient answers to the K‐SES_18_ and the ACL‐RSI for the pre‐surgery, 4‐, 8‐, 12‐ and 18‐months follow‐ups after ACL reconstruction were extracted from Project ACL in April 2024. The study sample consisted of a mixed cross‐sectional cohort at each follow‐up; some patients contributed data at multiple time points, while others responded at only one.

### Data management and statistical analysis

The ACL‐RSI is administered from 8 months after ACL reconstruction. The PASS and MCID were calculated in the included cohort at 4, 8, 12 and 18 months, while all follow‐ups were included in the calculation of the MIC.

Means, medians, standard deviations (SD) and ranges were reported for patient demographics. The PASS, MCID and MIC were calculated at different follow‐ups since the PROs aim to assess self‐reported symptoms, which can vary over time, e.g., the MCID for the K‐SES 8 months after ACL reconstruction could be different compared with 18 months after ACL reconstruction. Table 1 presents the definitions of PASS, MCID and MIC used in the present study, along with examples of their interpretation.

### Patient acceptable symptom state

After data extraction, patients were dichotomised at each follow‐up according to the answer to the first external anchor question as being satisfied with their knee function or not. Four separate analyses were performed for four different follow‐ups for the K‐SES_18,_ 4, 8, 12 and 18 months, and three for the ACL‐RSI, 8, 12 and 18 months. Consequently, three (ACL‐RSI) and eight (four for K‐SES_18_ present and four for K‐SES_18_ future) different PASS values were calculated respectively, depending on the time elapsed from ACL reconstruction. The PASS was determined with a receiver operating characteristic (ROC) curve [7]. A calculation for the area under the curve (AUC) was performed. The AUC is interpreted as the probability of identifying a patient who achieve PASS on the basis of the PRO score from randomly selected pairs of patients who achieve PASS and patients who do not achieve PASS. An AUC value of 0.7–0.8 is regarded as acceptable, and a value of 0.8–0.9 as excellent [13].

### Minimal clinical important difference

For clinically meaningful effects, MIC and MCID represent conceptually distinct metrics to interpret clinical relevance [9]. As semantically described, a change (MIC) occurs within a person, from one to another measurement point, while a difference (MCID) occurs between groups of people.

The MCID was calculated for each of the included follow‐ups: 4 months (K‐SES_18_ only), 8, 12 and 18 months. Consequently, three (ACL‐RSI) and eight (four for K‐SES_18_ present and four for K‐SES_18_ future) different MCID values were found, respectively, depending on the time elapsed from ACL reconstruction.

A minimum difference should be a difference between two adjacent levels of a scale. The MCID was defined as the difference in the change score of the 'improved' and 'non‐improved' patients [4]. Improved patients were defined as patients that answered, ‘agree’ or 'strongly agree' to the second anchor question, that is, 'do you think the treatment you received has markedly improved your condition?' Non improved were instead defined as patients that answered, 'neither agree nor disagree', 'disagree' or 'strongly disagree' to the same anchor question.

For calculation of the MCID, 10 methods have been presented [15]. For the purpose of this study, an anchor based method was chosen with the change difference (CD) method [15]. Accordingly, the MCID was identified by the difference between the average score change in ACL‐RSI and K‐SES_18_ respectively, of improved patients, defined by the anchor question, and the average score change of non‐improved patients.

### Minimal important change

The clinical use for a MIC is to interpret whether patients perceive their received treatment has improved their condition or not. Therefore, pre‐surgery answers to PROs were taken into consideration for MIC calculations. Time intervals in which MIC was calculated are presented in Table 2.

**Table 2:** Time intervals in which MIC was calculated.

Baseline PROs value	Referenced time point PROs value			
Pre‐surgery	4 months	8 months	12 months	18 months
4 months		8 months	12 months	18 months
8 months			12 months	18 months
12 months				18 months

Abbreviations: MIC, minimal important change; PROs, patient reported outcome measures.

Assuming that all patients have their individual threshold of what they consider a minimal important change, the MIC can be conceptualised as the mean of these individual thresholds. Accordingly, as explained by Terwee et al. [27], 'this definition of MIC is made up of three important elements: first, it refers to a threshold for a minimal change above which patients perceive themselves as changed (improved or deteriorated). Second, it refers to a change that is considered important to patients. And third, it refers to a within‐patient change over time' [27].

For the MIC an anchor‐based method was chosen, which involved anchoring the PRO change score to an external measure of important change, and the predictive modelling method, as the mean change and the ROC methods have been criticised for being subject to bias [8, 26, 27]. The predictive modelling approach is based on the predicted probability that a patient belongs to the improved group, based on the anchor, given the observed change score [26]. The predictive modelling approach implies logistic regression analysis with group variable, that is, improved or not improved on an anchor question as the dependent variable and the change score on the instrument of interest as the independent variable. The MIC value is then defined as the change score associated with a likelihood ratio of 1, that is, post‐test and pre‐test probability of belonging to the improved group are equal.

For the calculation of the MIC, the second anchor question was used: 'do you think the treatment you received has markedly improved your condition? (strongly disagree/disagree/neither agree or disagree/agree/strongly agree)', where 'agree' and 'strongly agree' were categorised as the improved group.

Statistical analyses were performed with the Statistical Analysis System (SAS) software 9.4 version (Copyright © 2013, SAS Institute Inc., Cary, NC, USA). A significance level of 0.05 was set.

## RESULTS

**Figure 1:**
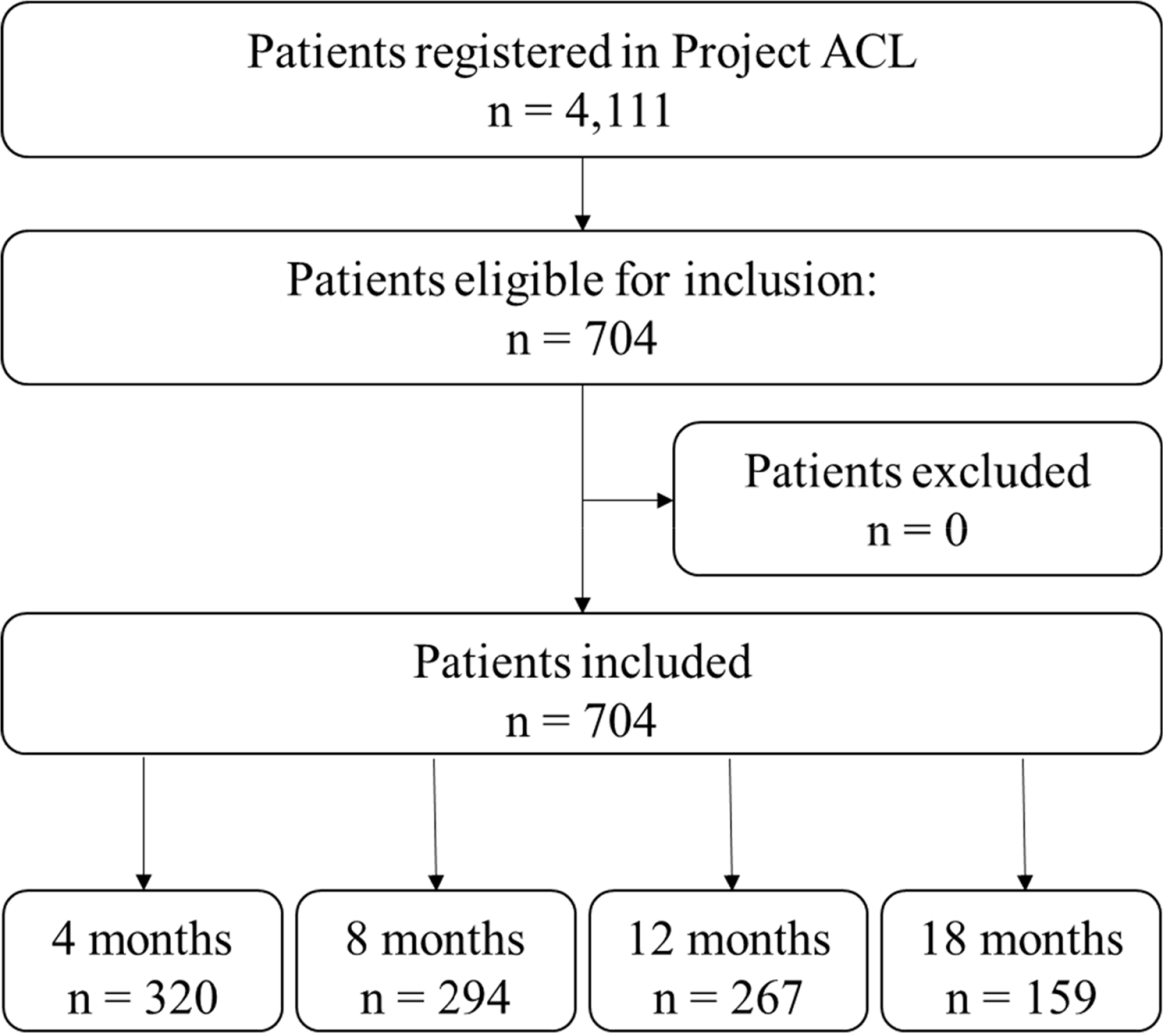
Flow chart of inclusion. The difference between the total number of patients in the registry and the included cohort reflects patients who did not meet the eligibility criteria for this study (e.g., missing PROs or anchor questions), not patients who were excluded after eligibility assessment. ACL, anterior cruciate ligament; PROs, patient reported outcome measures.

A total of 704 unique patients (58.4% male) were included in the study (Figure 1). Most patients registered in Project ACL were not eligible for inclusion in the present study, primarily due to missing responses on the ACL‐RSI and/or K‐SES_18_, or missing answers to the anchor questions required for calculation of PASS, MCID, and MIC. No patients were excluded after meeting the eligibility criteria. Included patients were on average 27.1 ± 9 years old at time of ACL reconstruction, and a majority of patients (82%) received hamstring tendon as autograft at reconstruction. Demographics of included patients, stratified by follow‐ups are presented in Table 3.

**Table 3:** Demographics of included patients at each follow‐up.

	4 months	8 months	12 months	18 months
Number of patients	320	294	267	159
Sex, male, *n* (%)	187 (58.4)	174 (59.2)	158 (59.2)	103 (64.8)
Age, years; mean (SD)	27.4 (8.9)	26.2 (8.6)	26.8 (9.0)	27.7 (9.5)
Height, cm; mean (SD)	173.0 (10.9)	174.1 (9.8)	174.0 (9.1)	172.6 (8.4)
Weight, kg; mean (SD)	72.1 (12.6)	72.5 (12.8)	71.9 (11.7)	71.6 (10.8)
BMI; mean (SD)	24.2 (6.3)	23.8 (2.8)	23.7 (2.8)	24.0 (2.7)
Graft				
Hamstring	82.1%	78.2%	75.4%	71.4%
Patella	15.5%	19.6%	22.2%	26.5%
Quadriceps	0.4%	0.4%	0.0%	0.0%
Other	2%	1.8%	2.4%	2.0%
Pre injury level of activity (Tegner)				
1–5	15%	13.2%	15.3%	14.4%
6	8.8%	7.2%	6.4%	7.5%
7	18.1%	18.0%	15.7%	18.2%
8	19.1%	21.4%	19.5%	13.8%
9	24.1%	28.6%	29.6%	30.2%
10	15.0%	11.6%	13.5%	15.7%

Abbreviations: BMI, body mass index; cm, centimeters; kg, kilograms; *n*, number; SD, standard deviation.

### Patient acceptable symptom state

The PASS was calculated with patients dichotomised as (1) satisfied, or (2) not satisfied with their knee function, according to the anchor question '*do you perceive your current physical status as satisfactory? (yes/no)*'. The proportion of patients who achieved PASS increased at each follow‐up (Table 4).

**Table 4:** PASS values for the K‐SES_18_ and the ACL‐RSI.

	K‐SES_18_ present			K‐SES_18_ future			ACL‐RSI		
	PASS values	AUC	Pass %	PASS values	AUC	Pass %	PASS values	AUC	Pass %
4 m	5.7	0.67	36%	7.5	0.65	36%			
8 m	7.7	0.71	37%	8	0.71	37%	56.6	0.67	37%
12 m	8.8	0.71	55%	8	0.68	55%	61.6	0.66	44%
18 m	8.2	0.82	71%	7	0.76	71%	65.5	0.79	70%

Abbreviations: ACL‐RSI, anterior cruciate ligament‐return to sport after injury scale; AUC, area under the curve; K‐SES_18_, 18‐item version of the knee self‐efficacy scale; m, months; *n*, number; PASS, patient acceptable symptom state; PROs, patient reported outcomes.

The AUC values were considered as acceptable [13] for the K‐SES_18_ present at 8, 12 and 18 months, the K‐SES_18_ future at 8 and 18 months, and for the ACL‐RSI at 18 months.

### MCID

The MCID was calculated between improved (IMP) and non‐improved (NIMP) patients. Improved patients were defined as patients that answered, 'agree' or 'strongly agree' to the second anchor question, that is, 'do you think the treatment you received has markedly improved your condition?' Non improved were instead defined as patients that answered, 'neither agree nor disagree', 'disagree' or 'strongly disagree' to the same anchor question. At each follow‐up 97% of the cohort was IMP and 3% was NIMP. Table 5 presents the MCID values for each follow‐up. The MCID values ranged between 1.6–4.1 for the K‐SES_18_, and 13.6–30.4 for the ACL‐RSI.

**Table 5:** MCID values between improved and non‐improved.

	4 months	8 months	12 months	18 months
K‐SES_18_ present				
IMP‐NIMP, %	97‐3%	97‐3%	97‐3%	97‐3%
MCID (95% CI)	2.3 (1.1–3.3)	2.0 (1.0–2.8)	2.9 (1.8–3.7)	3.9 (2.4–5.1)
K‐SES_18_ future				
IMP‐NIMP, %	97‐3%	97‐3%	97‐3%	97‐3%
MCID (95% CI)	2.1 (0.9–3.1)	2.2 (0.9–3.3)	1.6 (0.2–2.8)	4.1 (2.2–5.6)
ACL‐RSI				
IMP‐NIMP, %	n.a.	97‐3%	97‐3%	97‐3%
MCID (95% CI)	n.a.	13.6 (0.2–27.6)	16.3 (0.1–31.8)	30.4 (11.2–47.3)

Abbreviations: ACL‐RSI, anterior cruciate ligament‐return to sport after injury scale; CI, confidence interval; IMP, improved; K‐SES_18_, 18‐item version of the knee self‐efficacy scale; m, months; MCID, minimal clinical important difference; *n*, number; n.a., not applicable; NIMP, non‐improved.

### MIC

For the calculation of the MIC, patients who answered ‘neither agree or disagree’, ‘disagree’ or ‘strongly disagree’ with the statement “do you think the treatment you received has markedly improved your condition?” were defined as non‐improved. Patients who ‘agreed’ or ‘strongly agreed’ were defined as improved.

Table 6 presents MIC values for the K‐SES_18_ and ACL‐RSI. All MIC values for the K‐SES_18_ future except between 8 and 12 months, and all MIC values for the ACL‐RSI were negative. Negative MIC values suggest that the observed change scores were not associated with a clinically meaningful improvement and may reflect a lack of perceived progress rather than true deterioration.

**Table 6:** MIC values between different follow‐ups.

		Change to			
		4 m	8 m	12 m	18 m
	Change from	MIC (95% CI) [% improved: *n*]	MIC (95% CI) [% improved: *n*]	MIC (95% CI) [% improved: *n*]	MIC (95% CI) [% improved: *n*]
K‐SES_18_ present	Pre‐op	−0.2 (−0.9 to 0.4) [88% 76/86]	0.5 (−1.6 to 1.7) [95%: 72/76]	1.5 (0.1 to 2.8) [91%: 67/74]	2.1 (1.8 to 2.4) [91%: 42/46]
	4 m		1.1 (0.5 to 1.6) [94%: 168/178]	2.2 (1.3 to 2.7) [95%: 112/118]	2.1 (1.1 to 2.8) [96%: 50/58]
	8 m			0.7 (0.5 to 0.9) [96%: 150/157]	−0.3 (−3.7 to 0.6) [94%: 58/62]
	12 m				−0.8 (−2.3 to 0.2) [95%: 90/95]
K‐SES_18_ future	Pre‐op	−0.3 (−1.4 to 0.8) [88% 76/86]	−1.0 (−1.9 to −0.2) [95%: 72/76]	−0.3 (−1.0 to 0.5) [91%: 67/74]	−1.3 (−4.7 to 0.3) [91%: 42/46]
	4 m		−0.7 (−1.3 to −0.2) [94%: 168/178]	−0.5 (−1.3 to 0.1) [95%: 112/118]	−1.7 (−6.6 to 1.4) [96%: 50/58]
	8 m			0.0 (−0.6 to 0.6) [96%: 150/157]	−1.5 (−3.0 to −1.0) [94%: 50/58]
	12 m				−1.6 (−4.1 to −0.3) [95%: 90/98]
ACL‐RSI	8 m			−0.6 (−8.3 to 7.1) [95%: 147/154]	−1.5 (−12.1 to 7.8) [93%: 65/60]
	12 m				−1.1 (−9.0 to 7.4) [95%: 88/93]

*Note*: Since the proportion of patients improved is larger than 50%, the MIC values were adjusted for the proportion of improved patients. The ACL‐RSI is not administered pre‐operatively.

Abbreviations: ACL‐RSI, anterior cruciate ligament‐return to sport after injury scale; CI, confidence interval; K‐SES_18_, 18‐item version of the knee self efficacy scale; MIC, minimal important change; m, months.

## DISCUSSION

The main findings in this study were the different thresholds for the symptom state that patients perceived as acceptable (PASS), the minimum difference between patients who felt that treatment had improved their condition (MCID), and the minimal important change between follow‐ups for patients who felt that treatment had improved their condition (MIC) (see Tables 4, 5, 6). This study provides clinicians with practical thresholds to interpret scores on the ACL‐RSI and K‐SES_18_, and offers clinicians “benchmarks” to assess whether a patient's scores on those questionnaires reflect an acceptable state, and whether meaningful improvements have occurred over time (see Table 4). This should serve as an aid to tailor rehabilitation strategies and to help patients set realistic expectations throughout the recovery process.

Our findings support the importance of interpreting PROs using clinically relevant thresholds rather than relying solely on statistical significance [17]. As highlighted in recent literature [17], statistically significant differences may not always reflect patient‐relevant improvement. By reporting both MCID and MIC, and anchoring these thresholds in patient‐reported perception of improvement, we offer a more patient‐centred interpretation of the outcomes following ACL reconstruction. One of the present study's contributions is the demonstration that PASS, MCID, and MIC values are not static but evolve as patients move through different stages of recovery, which highlights the dynamic nature and the subjective assessment of patient's recovery. Previous studies, have proposed one value for PASS and MCID across different follow‐ups [8, 16]. The present study aligns with other studies and suggest that there are improvements in patients' subjective psychological and physical outcomes over time [2, 19, 24], which entails a need for time‐specific values to facilitate interpretation of PROs during rehabilitation after ACL reconstruction. However, it is important to recognise that the relevance of these outcomes may evolve as patients' priorities shift throughout their recovery. Importantly, the proportion of patients that achieved PASS and the proportion of patients that had improved their condition, that is, MIC, increased at each follow‐up from 4 to 18 months after ACL reconstruction. The increased proportion of patients who achieve the cut‐offs might suggest an overall trend of improvement in knee function and self‐efficacy, which is a positive indication of recovery progression. These findings suggest that rehabilitation timelines and clinical assessments must be tailored to the specific phase of recovery. In earlier follow‐ups, patients might experience persistent functional limitations or need to psychologically adapt to their injury, which may explain the lower proportion of PASS and MCID thresholds at 4 and 8 months after ACL reconstruction.

The AUC‐values associated with the calculated PASS scores were not above acceptable level for the K‐SES_18_ present at 4 months, the K‐SES_18_ future at 4 and 12 months, and the ACL‐RSI at 8 and 12 months, but indicated acceptable discrimination between satisfied and non‐satisfied patients at all other follow‐ups, particularly by 18 months' post‐surgery. Thus, the PASS scores could not distinguish between satisfied and dissatisfied patients at all follow‐ups. This inability to distinguish between patients could reflect one limitation with the use of PROs, that is, to evaluate in a snapshot of time an outcome that is subject to high variability, such as psychological response to an injury. Furthermore, the slight negative or near‐zero MIC‐values observed all MIC calculations for the K‐SES_18_ future subscale could indicate a plateau in self‐efficacy expectations, or possibly a recalibration of patient expectations with regard to future capabilities. This further underscores the importance to adapt rehabilitation to patient status, but also to adapt the interpretation of results from evaluation to patients' status [25].

The small proportion of patients categorised as non‐responders, could be interpreted both as a statistical limitation which is further described in the limitations section of this study, but also as a positive indication that most patients were satisfied with the received treatment, and perceived meaningful improvements in their condition. The low number of non‐responders suggests that the intervention overall was effective to address the physical and psychological needs measured by the included PROs of most patients, which led to favourable outcomes across follow‐ups. However, it is also possible that some patients adapted psychologically over time, adjusted expectations and redefined what constitutes improvement.

### Clinical implications

The result that MIC and MCID values increase over time, highlights the importance of rehabilitation strategies not only focused to the present moment, but also on longer‐term goals. The dynamic change in MIC, for instance, suggests that what patients consider a meaningful improvement in knee function at 8 months might differ from what they consider a meaningful improvement at 18 months. Thus, clinicians should continuously monitor patient goals and expectations to ensure meaningful recovery. The use of time‐specific PASS, MCID, and MIC values can offer a more accurate and individualised understanding of patient progress during rehabilitation. Specifically, early follow‐ups such as at 4 months also hold important clinical value. At this stage, many patients navigate the psychological and emotional challenges of rehabilitation, which can include for instance uncertainty about recovery or reduced self‐efficacy. To identify patients with low self‐efficacy or poor psychological adaptation early during rehabilitation allows clinicians to tailor rehabilitation strategies, offer targeted support, and potentially prevent long‐term psychological barriers. While the PASS thresholds reported here are specific to a Swedish cohort, the approach used to derive them can be applied across different settings. Clinicians and researchers working with other populations may use our findings as reference points or benchmarks and are encouraged to calculate their own population‐specific thresholds using similar methods.

### Limitations

This study is limited by the reliance on self‐reported outcomes, which are susceptible to biases such as response shift and recall bias [14]. Patients can place greater emphasis on their current postoperative state rather than the actual change when they respond to the anchor questions [23]. Anchor questions require patients to retrospectively assess how much their condition has improved and to judge the significance of that change. According to response shift theory, patients may adjust their internal standards to evaluate their condition over time, which could lead to paradoxical responses [10, 23]. With the response shift theory in mind, a patient may report a significant improvement in the condition even if the K‐SES_18_ present score remains unchanged. The reported improvement in spite of unchanged PROs score may occur due to recall bias, where patients struggle to accurately remember their preoperative state and instead base their judgement on their current situation [23]. The extent to which recall bias and response shift influenced results from the present study remains uncertain, but these factors could explain discrepancies between reported improvement and measured changes in PROs score. In addition, the proportion of patients categorised as non‐improved was small at some time points, which may have introduced instability in the MCID estimates due to imbalanced group sizes. While this limits the robustness of the between‐group comparison, the concurrent reporting of MIC provides a complementary and potentially more stable interpretation of meaningful change at the individual level. Future studies with larger groups of non‐responders, or patients who do not perceive the treatment to have improved their condition are warranted for replication of our results. The PASS thresholds reported in this study are specific to the Swedish population of patients undergoing ACL reconstruction and may not be directly generalisable to other populations or healthcare settings, where patient expectations and rehabilitation contexts may differ. Finally, the number of patients included varied across follow‐up points, and not all patients contributed data at every time point. This may introduce selection bias, as patients who responded at 12 or 18 months might differ in important ways (e.g., motivation and recovery status) from patients who did not. While this reflects real‐world registry‐based follow‐up, it should be acknowledged that our findings may overrepresent patients with more favourable outcomes or higher engagement in rehabilitation.

## CONCLUSION

The PASS, MCID and MIC values for the K‐SES_18_ and the ACL‐RSI vary over time. Practical thresholds are provided for clinicians to better interpret scores and to determine if meaningful improvements have been achieved.

## AUTHOR CONTRIBUTIONS

**Ramana Piussi**: Conceptualisation; methodology; formal analysis; investigation; data curation; writing–original draft; visualisation. **Jakob Lindskog**: Investigation; writing–original draft; visualisation. **Rebecca Hamrin Senorski**: Investigation; writing–original draft; visualisation. **Roland Thomeé**: Resources, writing–review and editing. **Kristian Samuelsson**: Resources, writing–review and editing. **Eric Hamrin Senorski**: Conceptualisation, methodology; formal analysis; investigation; writing–review and editing; supervision.

## CONFLICT OF INTEREST STATEMENT

Author Kristian Samuelsson is a board member of Getinge AB (publ). The remaining authors declare no conflicts of interest.

## ETHICS STATEMENT

Ethical approval for the project was obtained from the Swedish Ethical Review Authority (registration number: 2020‐02501) and the Regional Ethical Review Board in Gothenburg, Sweden (registration numbers: 265‐13, T023‐17). Informed written consent was obtained before participation in the study.

## Data Availability

Data are available from the corresponding author upon reasonable request.
